# The use of infrared thermography for non-invasive detection of bleeding and musculoskeletal abnormalities in patients with hemophilia: an observational study

**DOI:** 10.1186/s12959-023-00511-5

**Published:** 2023-06-28

**Authors:** Ryohei Kawasaki, Asuka Sakata, Chihiro Hosoda, Suguru Harada, Tetsuhiro Soeda, Yukiko Nishida, Naoki Matsumoto, Kohei Tatsumi, Keiji Nogami, Yasushi Yoshimura, Midori Shima

**Affiliations:** 1 Medicinal Biology of Thrombosis and Hemostasis, Nara Medical University, 840 Shijo-Cho, Kashihara, Nara 634-8521 Japan; 2 Product Research Department, Medical Affairs Division, Chugai Pharmaceutical Co., Ltd., Yokohama, Japan; 3 Advanced Medical Science of Thrombosis and Hemostasis, Nara Medical University, Kashihara, Japan; 4 Project Planning and Coordination Department, Translational Research Division, Chugai Pharmaceutical Co., Ltd., Chuo-ku, Japan; 5 Department of Pediatrics, Nara Medical University, Kashihara, Japan

**Keywords:** Bleeding, Hemophilia, Injury, Skin temperature, Thermography

## Abstract

**Background:**

In patients with hemophilia (PwH), bleeding often occurs in joints and muscles, and early detection of hemorrhage is important to prevent the onset and progression of mobility impairment. Complex-Image analysis such as ultrasonography, computed tomography, and magnetic resonance imaging are used to detect bleeding. On the other hand, no simple and rapid method to detect the active bleeding has been reported. Local inflammatory responses occur when blood leaks from damaged vessels, and the temperature at the site of active bleeding could be expected to increase in these circumstances, leading to an increase in surrounding skin temperature. Therefore, the purpose of this study was to investigate whether the measurement of skin temperature using infrared thermography (IRT) can be used as a diagnostic aid to detect active bleeding.

**Methods:**

Fifteen PwH (from 6 to 82 years old) complaining of discomfort such as pain were examined. Thermal images were obtained simultaneously at the affected sides and comparable unaffected sides. The average skin temperature of the affected side and of the unaffected side were measured. The temperature differences were calculated by subtracting the average skin temperature at the unaffected side from the affected side.

**Results:**

In eleven cases with active bleeding, the skin temperature at the affected side was more than 0.3 °C higher (0.3 °C to 1.4 °C) compared to the unaffected side. In two cases without active bleeding, there were no significant differences in skin temperature between the affected and unaffected sides. In two cases with previous rib or thumb bone fracture, the skin temperature at the affected side was 0.3 °C or 0.4 °C lower than that of the unaffected side, respectively. In two cases with active bleeding in which longitudinal evaluation was conducted, the difference in skin temperature decreased after hemostatic treatment.

**Conclusion:**

The analysis of skin temperature deference using IRT was a useful supportive tool to readily assess musculoskeletal abnormalities and bleeding in PwH as well as to determine the success of the hemostatic treatment.

**Supplementary Information:**

The online version contains supplementary material available at 10.1186/s12959-023-00511-5.

## Background

Hemophilia is an X-linked recessive bleeding disorder caused by a defect or abnormality of factor VIII (hemophilia A) or factor IX (hemophilia B) [1, 2]. Patients with hemophilia (PwH) commonly bleed within muscles and joints, not only after injury but also during physical exercise or normal daily activities [3]. Intramuscular bleeding has a significant impact on daily life, ranging from short-term impairment, such as muscle deconditioning and compartmental syndromes, to longer-term problems including muscle atrophy with abnormal body balance and further injuries [4]. Repeated joint hemorrhages lead to synovial proliferation, neo-angiogenesis, and ultimately, damage to both the articular cartilage and subchondral bone, resulting in the condition known as hemophilic arthropathy [5, 6]. Consequently, PwH with hemophilic arthropathy suffer from chronic pain, muscle and joint dysfunction, and limited mobility. Early detection of bleeding and adequate prompt treatment is central, therefore, to maintaining the quality of life of these individuals.

Recent advances in hemophilia treatment, including the widespread use of prophylactic replacement therapy with coagulation factor agents, extended half-life coagulation factor concentrates, and non-factor agents, have led to a decrease in the annual bleeding rates (ABRs) [7–9]. In clinical studies of joint function, those PwH treated with prophylactic FVIII agents had less symptomatic bleeding and better joint conditions than those receiving episodic therapy with FVIII agents [10, 11]. Nevertheless, some patients continue to develop arthropathy that leads to mobility impairment, maybe because the symptoms of bleeding are less severe and not immediately apparent.

The diagnosis of hemorrhage into muscles and joints in PwH often depends on imaging procedures such as ultrasonography, computed tomography (CT), and magnetic resonance imaging (MRI) [12]. MRI appears to be the most sensitive method for determining the presence or absence of hemorrhage, although its routine use is restricted due to high cost and the length of time required for the examination. In addition, paediatric patients often require sedation which can limit the interpretation of image data. Ultrasonography, which provides a good technique at point-of-care, is less expensive and is technically more easily accessible than MRI, but is also time consuming and dependent on highly skilled operators. A major disadvantage of CT relates to need for radiation-based images [12].

In recent years, infrared thermography (IRT) has also been introduced into medical practice, and facilitates detection of physical abnormalities and injury by measuring side-to-side differences in skin temperature. Asymmetry of more than 0.5 °C is not physiologic in non-hemophilic healthy individuals [13, 14]. Seuser et al. utilised IRT combined with clinical examination using a Hemophilia Joint Health Score (HJHS) in children with hemophilia and age-matched healthy boys [15]. Their data suggested that IRT detected more signs of an early inflammatory response in the PwH than in healthy children. Local inflammatory responses occur when blood leaks from damaged vessels, and the temperature at the site of active bleeding could be expected to increase, leading to the rise of surrounding skin temperature [16]. Measurements of changes in skin temperature using IRT may, therefore, help to detect active bleeding. To the best of our knowledge, however, the use of IRT for localizing active subcutaneous and musculoskeletal bleeding in PwH remains to be investigated.

In the present study, we have investigated whether the measurement of the change in skin temperature using IRT may offer a relatively straightforward diagnostic aid to determine the presence or absence of active bleeding. In addition, we assessed temperature changes over time after the initiation of hemostatic treatment.

## Methods

### Subjects

Fifteen male children and adults with hemophilia (from 6 to 82 years old) enrolled from November 2019 to December 2021 were examined using IRT on admission to Nara Medical University Hospital with complaints of discomfort such as pain, in skin, joints, or muscles. The presence of active bleeding was also examined using ultrasonography or MRI, after symptomatic relief following administration of coagulation factor concentrates. Assessments were performed bilaterally, at the affected side as the objective area and on the comparable unaffected side as the control area. The study was conducted after obtaining informed consent, and were approved by the Nara Medical University Ethics Committee and Chugai Ethics Committee.

### Thermal image acquisition by IRT

Thermal images were obtained using two IRT systems. The Vision Sensing system (Vision Sensing Co., Ltd, Osaka, Japan), utilized a spectral sensitivity ranging from 8 µm to 14 µm and a geometric resolution of 2.476 mrad (640 × 480 pixels focal plane array and a 90.8° field of view lens, with a minimum focus distance approximately 45 cm). The FLIR system (Teledyne FLIR LLC, Wilsonville, Oregon, United States), employed a spectral sensitivity ranging from 7.5 µm to 14 µm and a geometric resolution of 1.75 mrad (320 × 240 pixels focal plane array and a 23.65° field of view lens with a minimum focus distance approximately 15 cm). Prior to taking thermal images, patients removed their clothes so that affected side and unaffected side were exposed and allowed their skin to accustom to the surrounding temperature for at least 5 min. During taking thermal images, air conditioner wind and sunlight were kept away from the patients to minimize the influence of the environment.

### Analysis

Thermal images acquired using the Vision Sensing system were analyzed using ImageJ software (National Institutes of Health, MD, USA) to determine the average brightness values in the regions of interest (ROI) at the affected sides and the unaffected sides. ROI were based on the articular and muscular areas of human anatomy around the affected and the unaffected areas, by reference to previous studies [17]. The average temperatures in ROI were calculated from the average brightness values using the following formula provided by Vision Sensing Co. SiTF in the formula stands for Signal Transfer Function, which value represents the brightness change per 1 °C of temperature change on the image. Btemp is the temperature value at which the reference brightness value (8192) is determined during calibration in this system.

$$\begin{array}{c}average\, temperature= \frac{average\, brightness\, value-8192}{SiTF}+Btemp\\ SiTF=100, Btemp=50\end{array}$$

Thermal images acquired using the FLIR system were analyzed using Thermal Studio Pro (Teledyne FLIR LLC, Wilsonville, Oregon, United States) to determine the average skin temperature in the ROI at the affected sides and the unaffected sides. ROI were based on the articular and muscular areas of human anatomy as described above [17].

Absolute temperature is affected by various factors such as age, hair density, and/or genetic, as well as severity and location of bleeding [14, 18]. For this reason, the absolute temperature was not compared between subjects. Instead, the relative temperature of the affected side to the unaffected side in the same subject was evaluated. The differences in skin temperature (Δ skin temp) were calculated by subtracting the average skin temperature at the unaffected side from that of the affected side.

## Results

**Fig. 1:**
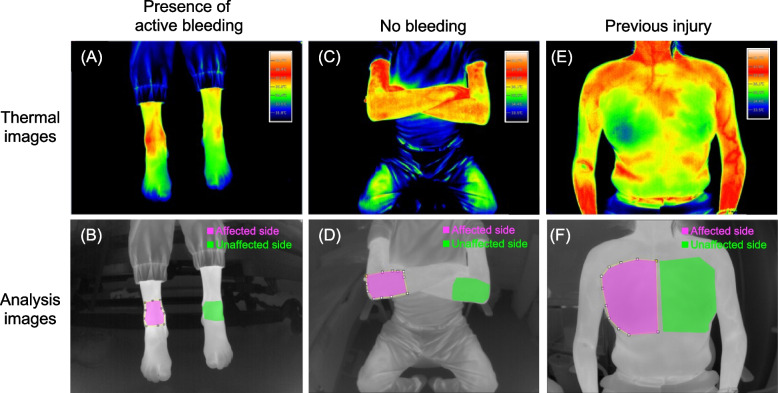
Thermal and analytical images using the Vision Sensing system. Representative images of active bleeding in Subject 3 (**A**, **B**), no bleeding in Subject 12 (**C**, **D**), and previous injury in Subject 14 (**E**, **F**). Areas coloured in magenta illustrate the affected side where bleeding or pain occurred. Areas coloured in green illustrate the unaffected, opposite side

Representative thermal and analytical images acquired using the Vision Sensing system are shown for a patient with active bleeding into the right ankle joint (Subject 3), a patient with pain in the right elbow without bleeding (Subject 12), and a patient with a previous rib bone fracture (Subject 14) (Fig. 1). Upper panels illustrate thermal images (Fig. 1A, C, and E), and lower panels represent analytical images (Fig. 1B, D, and F). The areas coloured in magenta indicate the ROI of the affected side of the body and the areas coloured in green indicate the ROI of the unaffected side (Fig. 1B, D, and F). For the evaluation of thermal images, Δ skin temp was calculated by subtracting the average skin temperature at the unaffected side (areas coloured in green) from that of the affected side (areas coloured in magenta). The Δ skin temps were + 0.9 °C, 0 °C, and –0.3 °C in Subject 3, Subject 12, and Subject 14, respectively (Fig. 1 and Table 1).

**Table 1:** Summary of skin temperature analysis and clinical information in hemophilia patients

**Subject**	**Severity of hemophilia**	**Age (years)**	**Days from injury or discomfort to taking image**	**Affected location**	**Bleeding**	**Thermography System**	**Δ skin temp**^**a**^	**Category**
1	Mild Hemophilia A	82	5	Dorsal surface of right thigh	Subcutaneous Active bleeding	Vision Sensing	+1.4 °C	Increase in temperature +0.3 °C or more
2	Mild Hemophilia A	11	7	Right ankle	Intra-articular Active bleeding	Vision Sensing	+0.9 °C	
3	Mild Hemophilia A	11	1	Right ankle	Intra-articular Active bleeding	Vision Sensing	+0.9 °C	
4	Mild Hemophilia B	12	1	Right ankle	Intra-articular Active bleeding	FLIR	+0.8 °C	
5	Mild Hemophilia A	19	23	Left intestinal muscle	Intramuscular Active bleeding	FLIR	+0.5 °C	
6	Mild Hemophilia A	45	1	Left lower leg inner side	Intramuscular Active bleeding	FLIR	+0.5 °C	
7	severe Hemophilia B	6	7	Dorsal surface of right thigh	Subcutaneous Active bleeding	FLIR	+0.5 °C	
8	Mild Hemophilia A	12	31	Right ankle	Intra-articular Active bleeding	FLIR	+0.4 °C	
9	Severe Hemophilia A	16	Unknown	Left lower leg inner side	Intramuscular Active bleeding	Vision Sensing	+0.4 °C	
10	Severe Hemophilia A	11	7	Left knee	Intra-articular Active bleeding	FLIR	+0.3 °C	
11	Mild Hemophilia A	16	1	Left iliopsoas muscle	Intramuscular Active bleeding	Vision Sensing	+0.3 °C	
12	Severe Hemophilia A	61	Unknown	Right elbow	No bleeding	Vision Sensing	0 °C	No difference in temperature
13	Severe Hemophilia A	18	2	Left knee	No bleeding	Vision Sensing	0 °C	
14	Severe Hemophilia A	49	18	Right chest	No bleeding (Previous fractured ribs)	Vision Sensing	–0.3 °C	Decrease in temperature –0.3 °C or more
15	Mild Hemophilia A	21	4	Right proximal phalanx of thumb	No bleeding (Previous fractured thumb)	FLIR	–0.4 °C	

^a^Difference in temperature between the affected side and the unaffected side

**Fig. 2:**
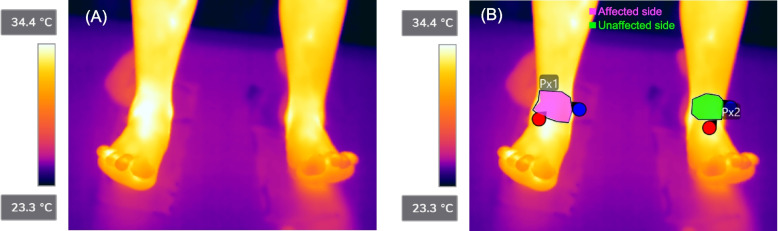
Representative thermal and analytical images using the FLIR system. Representative images of active bleeding in Subject 4 (**A**, **B**). Areas coloured in magenta illustrate the affected side where bleeding or pain occurred. Areas coloured in green illustrate the unaffected, opposite side

Representative thermal images and corresponding analytical images acquired using the FLIR system are shown for a patient with active bleeding into the right ankle joint (Subject 4) (Fig. 2). The Δ skin temp was + 0.8 °C in Subject 4 (Fig. 2 and Table1).

The characteristics and the results of IRT analysis in all cases are shown in Table 1. Thermal images were obtained from two cases of subcutaneous bleeding (Subject 1 and 7), five cases of intra-articular bleeding (Subject 2, 3, 4, 8, and 10), four cases of intramuscular bleeding (Subject 5, 6, 9, and 11). In cases with active bleeding (Subject 1–11), Δ skin temps were + 1.4 °C, + 0.9 °C, + 0.9 °C, + 0.8 °C, + 0.5 °C, + 0.5 °C, + 0.5 °C, + 0.4 °C, + 0.4 °C, + 0.3 °C, and + 0.3 °C (mean + 0.6 °C and median + 0.5 °C). These data identified skin temperatures of the affected side more than + 0.3 °C compared with the unaffected side in individuals with active bleeding. In Subject 12 and Subject 13, without evidence of active bleeding, there were no difference in skin temperature between the affected side and unaffected side. In Subject 13, who also had a limited range of motion in the right ankle without pain or other subjective symptoms, Δ skin temp was –0.7 °C (Supplementary Fig. 1). In Subject 14, who had a previous history of a right rib bone fracture, Δ skin temp was –0.3 °C. In Subject 15, who had a previous history of a right thumb bone fracture, Δ skin temp was –0.4 °C. These findings indicated, therefore, that a difference of 0.3 °C or more between affected and unaffected sides could categorise cases into those with active bleeding or those with past injury (Table 1).

**Fig. 3:**
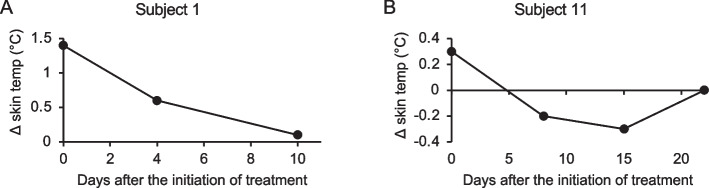
Changes in Δ skin temp after the initiation of treatment. **A** Changes in Δ skin temp after the initiation of treatment in Subject 1 with subcutaneous bleeding on the dorsal surface of right thigh. The patient was admitted to the hospital from the day treatment began and was discharged 10 days later. **B** Changes in Δ skin temp after the initiation of treatment in Subject 11 with intramuscular bleeding in the left iliopsoas muscle. The patient was admitted to the hospital from the day treatment began and discharged 15 days later. In Subject 11, rehabilitation of the right leg muscle via strengthening and stretching was started on Day 1. Rehabilitation of the left foot via loading and gait training with crutches was started on Day 7

Subject 1 and Subject 11 required prolonged hospitalization for active bleeding, which allowed us for further longitudinal image analyses during admission. In Subject 1 on the first day of hospitalization, Δ skin temp was + 1.4 °C (Table 1). The patient was discharged 10 days after effective hemostatic treatment, and at this time Δ skin temp had decreased to + 0.1 °C (Fig. 3A). In Subject 11 on the first day of hospitalization, Δ skin temp was + 0.3 °C (Table 1). Δ skin temp was –0.2 °C 8 days after the hemostatic treatment, and was –0.3 °C on Day 15. In this patient, physiotherapy was commenced on Day 1 to strengthen the right leg muscle (unaffected side), and further rehabilitation of the left leg (affected side) with loading and gait training with crutches was commenced on Day 7. On Day 22, there was no difference in skin temperature between the affected side and the unaffected side (Fig. 3B).

## Discussion

Our results demonstrated that the evaluation of skin temperature difference using IRT might provide important diagnostic support for detecting active bleeding in PwH. In addition, the findings suggested that monitoring using IRT could be useful for determining the duration of hemostatic treatment and the effectiveness of rehabilitation.

Our IRT analysis systems discriminate temperature differences of 0.15 °C (Vision Sensing) and 0.04 °C (FLIR), and our data indicated that Δ skin temp of + 0.3 °C or more between the affected side and the opposite unaffected side distinguished active bleeding from no bleeding (Table 1). We observed some cases with slight increase in skin temperature (Table 1, Subject 10 and 11). It has been reported that the degree of increase in skin temperature after injury (acute phase) appears to vary in different anatomical locations [14]. In addition, bleeding may stop spontaneously after certain days from discomfort, which may affect to Δ skin temp. The results in these patients along with the previous reports indicated that careful observations of skin temperature could help along with the clinical management when the patient complains of discomfort or pain. The previous studies were designed to investigate acute phase injuries including muscle damage, sprain and bone fracture in non-hemophilic individuals and demonstrated a mean temperature difference of > 0.5 °C between affected and opposite, unaffected areas [14]. Minor bleeding that resolves spontaneously may be largely inconsequential in healthy individuals, but can lead to further orthopaedic difficulties in untreated PwH. Our definition of a lower detection limit (≧ 0.3 °C), therefore, could inform diagnosis of non-overt active, intramuscular bleeding in this clinically demanding group of patients.

Recent progress with hemophilia therapy, including the widespread use of prophylactic replacement therapy with coagulation factor agents and the development of the novel non-factor agent, emicizumab [7–9], has led to a marked reduction in ABRs. In a recent phase 2 study, patients with severe PwH A treated with emicizumab reported that the symptoms appeared to have changed to that of a milder phenotype, that the duration of bleeding was shortened, and that their emotional state was improved [19]. In these circumstances, therefore, the presence or absence of active bleeding could be difficult to determine, and evaluation of skin temperature using IRT might be especially useful. Currently, doctors have to take thermal images and analyse them, but in the future, as analysis technology develops and automatic diagnosis becomes available, patients and their families may be able to carry out assessments without visiting the hospital. Analysis of skin temperature may also be useful in confirming the effectiveness of hemostatic treatment. At present, clinical judgements on the need for continued replacement therapy might tend to be empirical, and our results indicated that Δ skin temp analysis could provide more objective data. For example, Subject 3 attended hospital one day after injury, and Subject 2 sought medical attention one week after trauma. Nevertheless, increases in skin temperature of the affected side were observed, suggesting prolonged or recurrent bleeding. In contrast, the elevated skin temperatures on the affected side in Subject 1 and Subject 11 recovered, and the subcutaneous hematoma were resolved, reflecting the success of hemostatic treatment (Fig. 3A and B).

Furthermore, IRT may be useful not only for assessing the duration of hemostatic treatment, but also for detecting a decline in physical function and the need for and effectiveness of rehabilitation. In Subject 14 with a previous rib bone fracture and Subject 15 with previous thumb bone fracture, the skin temperature was decreased on the affected side at the time of visit (Table 1). In a case study, Auf der Strasse, et al. reported that skin temperatures at an injured area were decreased after tibial fracture, and were then restored as the bone recovered [20]. Similarly, Park et al. reported that the skin temperatures on the affected side of patients with shoulder impingement syndrome were depressed in comparison with those at the unaffected side [21]. In our Subject 13, there were no subjective symptoms, but the skin temperature around the right ankle was 0.7 °C lower than that of the left ankle (Supplementary Fig. 1). He had a limited range of motion in the right ankle without obvious changes on X-ray. The thermography images in this case demonstrated that thermography detected hemophilic arthropathy in advance of conventional radiographic evidence. In addition, previous reports have indicated that decreased skin temperature at affected sides might reflect subacute injury or impaired physical function [20, 21]. In our Subject 11, the skin temperature of the affected side fell below that of the unaffected side after hemostatic treatment (Fig. 3B), and Δ skin temp gradually diminished after the initiation of rehabilitation. This suggests, therefore, that IRT could also be useful for determining the effectiveness of rehabilitation, and that in cases not needing urgent treatment, further medical examination could be desirable if Δ skin temp is ≦ –0.3 °C.

There are limitations to the present study. ABRs in PwH have decreased in recent years [22] and the number of suitable cases available for investigation was small. Also, the effects on skin temperature of inflammation in the absence of bleeding, temperature changes immediately after injury and bleeding, and blood volume contained at the injured site have not been examined. When inflammation occurs, an increase in temperature is expected even if the absence of bleeding. However, if there is an increase in temperature, whether it is bleeding or inflammation from other causes, the patient may require a detailed examination. Further investigations are being devised to establish the detailed relationship between these physical functions and temperature changes.

## Conclusions

The analysis of Δ skin temp using IRT was found to be a useful supportive tool to readily assess musculoskeletal abnormalities and bleeding in PwH. In addition, the continuous analysis of Δ skin temp in individual PwH appeared to help determine the success of hemostatic treatment, the duration and effectiveness of rehabilitation and the detection of early phases hemophilic arthropathy sooner than conventional radiography.

## Supplementary Information

**Additional file 1.** Representative thermal imageand analytical imageassociated with a limited range of motion in right ankle in Subject 13. Areas coloured in magenta illustrate the affected side. Areas coloured in green illustrate the healthy side opposite to the affected side.

**Additional file 2.** Specification of infrared thermography.

## Data Availability

The datasets used/or analyzed during the current study are available from the corresponding author on reasonable request.

## Abbreviations

ABRs: Annual bleeding rates
CT: Computed tomography
HJHS: Hemophilia Joint Health Score
IRT: Infrared thermography
MRI: Magnetic resonance imaging
PwH: Patients with hemophilia
ROI: Regions of interest
Δ skin temp: Difference in skin temperature
