# Supplementary figures and images for: The use of infrared thermography for non-invasive detection of bleeding and musculoskeletal abnormalities in patients with hemophilia: an observational study
Source: Thromb J. 2023 Jun 28;21:70. doi: 10.1186/s12959-023-00511-5 (PMC10303282; doi:10.1186/s12959-023-00511-5)

Thermal image

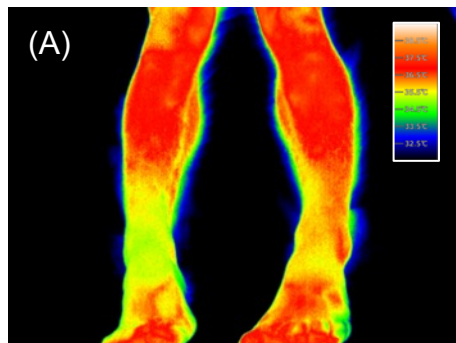

Analysis image

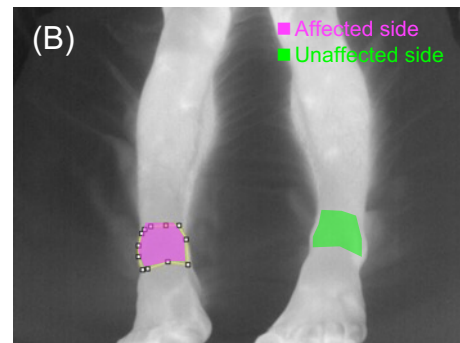

Supplement: Supplementary file 1 — Additional file 1. Representative thermal imageand analytical imageassociated with a limited range of motion in right ankle in Subject 13. Areas coloured in magenta illustrate the affected side. Areas coloured in green illustrate the healthy side opposite to the affected side. [file 12959_2023_511_MOESM1_ESM.pdf]
