# Supplementary material for: The use of infrared thermography for non-invasive detection of bleeding and musculoskeletal abnormalities in patients with hemophilia: an observational study
Source: Thromb J. 2023 Jun 28;21:70. doi: 10.1186/s12959-023-00511-5 (PMC10303282; doi:10.1186/s12959-023-00511-5)
Supplement: Supplementary file 2 — Additional file 2. Specification of infrared thermography. [file 12959_2023_511_MOESM2_ESM.docx]

Supplementary Information

**Specification of infrared thermography**

Thermal sensitivity was 0.15°C and absolute temperature measurement accuracy was approximately ±1°C in the Vision Sensing system. Thermal sensitivity was 0.04°C and absolute temperature measurement accuracy was approximately ±0.3°C in the FLIR system. The absolute temperature measurements with these procedures had tolerance limits between images up to 1°C or 0.3°C, respectively, but differences of 0.15°C or 0.04°C, respectively, could be distinguished within the same image. We provide an example for sensitivity and absolute temperature. Sensitivity is the identifiable temperature difference between adjacent pixels in the same image. Regarding absolute temperature, when an object is taken images several times, the absolute temperature in each image will vary even for the same object. In the same image, the relative temperatures are not inverted, and the temperature difference of 0.3°C (the cut line set in this study) can be identified with the systems we used. Therefore, thermal images were obtained simultaneously at the affected side and the opposite unaffected side.
